# Investigation of Machine Learning Approaches for Traumatic Brain Injury Classification via EEG Assessment in Mice

**DOI:** 10.3390/s20072027

**Published:** 2020-04-04

**Authors:** Manoj Vishwanath, Salar Jafarlou, Ikhwan Shin, Miranda M. Lim, Nikil Dutt, Amir M. Rahmani, Hung Cao

**Affiliations:** 1 Department of Electrical Engineering and Computer Science, University of California, Irvine, CA 92607, USA; manojv@uci.edu (M.V.); ikhwans@uci.edu (I.S.); dutt@ics.uci.edu (N.D.); 2 Erik Jonsson School of Engineering and Computer Science, Dallas, TX 75201, USA; salarjafarlou@gmail.com; 3 VA Portland Health Care System, Portland, OR 97239, USA; lmir@ohsu.edu; 4 Departments of Neurology, Behavioral Neuroscience, Medicine, and Oregon Institute of Occupational Health Sciences, Oregon Health & Science University, Portland, OR 97239, USA; 5 Department of Computer Science, University of California, Irvine, CA 92607, USA; a.rahmani@uci.edu; 6 School of Nursing, University of California, Irvine, CA 92607, USA; 7 Department of Biomedical Engineering, University of California, Irvine, CA 92607, USA

**Keywords:** electroencephalogram (EEG), machine learning (ML), traumatic brain Injury (TBI)

## Abstract

Due to the difficulties and complications in the quantitative assessment of traumatic brain injury (TBI) and its increasing relevance in today’s world, robust detection of TBI has become more significant than ever. In this work, we investigate several machine learning approaches to assess their performance in classifying electroencephalogram (EEG) data of TBI in a mouse model. Algorithms such as decision trees (DT), random forest (RF), neural network (NN), support vector machine (SVM), K-nearest neighbors (KNN) and convolutional neural network (CNN) were analyzed based on their performance to classify mild TBI (mTBI) data from those of the control group in wake stages for different epoch lengths. Average power in different frequency sub-bands and alpha:theta power ratio in EEG were used as input features for machine learning approaches. Results in this mouse model were promising, suggesting similar approaches may be applicable to detect TBI in humans in practical scenarios.

## 1. Introduction

Traumatic brain injury (TBI) is a common cause of disability and death in young people [1]. Caused by external impact such as blunt trauma, penetrating objects or blast waves to the head, TBI is becoming increasingly prevalent with an estimated 1.6 million individuals sustaining mild traumatic brain injury (mTBI) each year. Major causes of TBI have been vehicle related collisions, sports or combat injuries causing brain damages, including tearing injuries of white matter or hematomas resulting in nausea, disturbed sleep patterns [2], dizziness, memory and/or concentration problems, emotional disturbances and seizures. Many of whom are never hospitalized and may suffer from consequences of head injury. Lack of consensus regarding what constitutes mTBI adds to the complication of the under-diagnosis of the disease [3]. The recent advances in electroencephalogram (EEG) acquisition as well as quantitative electroencephalogram (qEEG) analysis have enabled a host of practical applications, such as detection of TBI in the field, making it more convenient compared to the counterpart of using bulky and resource-intensive CT scans. The need for high-end lab equipment to acquire CT scans eliminates its use in field-capable detection of TBI. Due to volume averaging in CT, there are possibilities of missing small amounts of blood that occupy widths less than a slice. Performance of automatic diagnostics through machine learning technique is comparable with traditional methods based on the visual inspection of CT scans and review of a patient’s symptoms [4]. As a result of the limitations of existing methods used to detect TBI, there is a need for new technology capable of rapid, accurate, non-invasive, and field-capable detection of TBI to bridge the technological gap that exists today. More rapid and accurate detection and measure of mTBI would lead to improved prognoses and minimize further impacts and complications during the event.

Electroencephalogram (EEG) is the physiological recording of electrical activity of the brain. It is used to record the electrical potential difference between two electrode positions compared to a reference electrode. This potential difference reflects synchronous firing of a population of neurons thereby recording diffused activity of the brain with high temporal resolution. Classical EEG oscillations range from below 1 Hz to around 100 Hz which have been extensively studied for various medical applications. These classical frequency bands include delta (0.5–4 Hz), theta (4–8 Hz), alpha (8–12 Hz), beta (13–30 Hz), and gamma (30–100 Hz) [5], each of which exhibits unique underlying physiological mechanisms. EEG is used to evaluate several brain disorders such as epilepsy, Alzheimer’s disease and lesions of the brain which reveals as seizure or unusually slow waves in EEG. EEG is assessed by clinicians for quality of the recording and other clinical interpretations such as presence of pathological potentials mentioned previously, which may result in inter-observer variability. Hence, to obtain more reliable assessment of EEG signals, the introduction of quantitative analysis is critical.

Clinical assessment of mTBI lacks robust objective markers; however, evidence suggests that TBI causes changes to EEG. Previous studies on mTBI primarily focused on spectral power and feature-driven approaches such as cross-frequency coupling using qEEG analyses. These studies gave promising results with increased delta [6,7] within different sleep stages [6,8] and attenuated alpha activity [9] which is used to detect the altered electrical activity in the brain. Our team has previously established persistent changes in the sleep pattern in mice following fluid percussion injury (FPI) which also shed light into time spent in different sleep stages and inability to maintain continuous bouts of wakefulness [6]. Most of the previous studies have concentrated on the changes in different frequency sub-bands such as enhanced beta power during non-rapid eye movement (NREM) sleep [8]. While most results are somewhat varied due to inconsistency in electrode placement, sample size, misclassification between different levels of TBI, they all focus on band power and can be generalized with a decrease in alpha band power and an increase in theta band power. A number of review studies has been conducted to study the progress of mTBI investigations using qEEG [10,11].

Machine learning approaches such as support vector machine (SVM), decision tree (DT), artificial neural network (ANN) have also been used to classify TBI [4,12]. Most existing machine learning models use inputs from various brain imaging techniques to classify TBI patients. In contrast, here, we directly use EEG for classification as the ultimate goal is to build a portable system which will be capable of detecting mTBI via EEG assessment in a field-based scenario.

In this study, we use EEG data acquired from a compelling mouse model of mTBI, lateral fluid percussion injury (FPI), which demonstrates very similar behavioural deficits and pathology to those found in humans suffering from mTBI, including sleep disturbances [6,13] to explore performance of various widely-used machine learning algorithms. EEG sub-band power acts as feature vectors in our study for rule-based machine learning algorithms however, convolutional neural network (CNN) considers temporal dynamics of raw EEG to automatically extract features suitable for classification.

## 2. Materials and Methods

### 2.1. Animal Data

Animal data were acquired as part of a previously published dataset that analyzed other sleep measures not described in this novel analysis [6,7]. Animal experiments were performed on 10-week-old, 25 g, male C57BL/6J mice (Jackson Laboratory) which were housed in a room maintained at ambient temperature of 23±1 °C with a relative humidity of 25±5% and automatically controlled 12-h light/12-h dark cycle illumination intensity 100 lx. The animals had free access to food and water. Animal experiments were performed in accordance with the guidelines published in the National Institutes of Health Guide for the Care and Use of Laboratory Animals and approved by the local IACUC committee.

### 2.2. Fluid Percussion Injury (FPI) and EEG/Electromyography (EMG) Sleep-Wake Recordings

**Figure 1:**
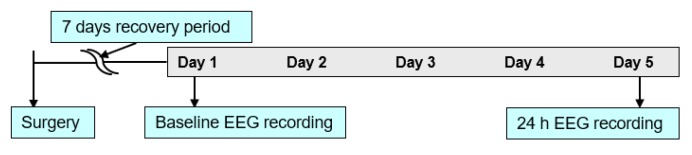
Experimental procedure for electroencephalogram (EEG) studies.

Fluid percussion injury in combination with EEG/EMG implantation in mice was performed as previously described in Reference [6]. Animals were divided into two groups: TBI and sham. Craniotomy was conducted over the right parietal area between bregma and lambda. The location was just medial to the sagittal suture and lateral to the lateral cranial ridge and a rigid needle hub was secured to the skull. The animal was anesthetized briefly, prior to the removal of the cap over the hub in the next day. Once the hub was fitted with the FPI device and monitored till the stage of toe pinch withdrawal reflex, a 20-ms pulse of saline was delivered onto the dura with pressure level reading between 1.4 and 2.1 atm. This has previously been shown by various research groups to induce mTBI [14,15]. The hub was removed from the skull immediately. Sham controls underwent the procedure mentioned above with an exception of fluid pulse. Mice were connected to a recording cable 5 days after the 7 day recovery period. Once the animal acclimatized, recording was initiated after 24 h. To ensure stable sleep/wake activity across days baseline sleep was analyzed on the first and fifth days [7]. The experimental timeline is shown in Figure 1.

### 2.3. EEG and Sleep/Wake Scoring

The 24-h recording with a sampling rate of 256 Hz obtained from each animal was scored by an experienced and blinded scorer into 4 s epochs of wake (W), non-rapid eye movement (NREM) and rapid eye movement (REM) as previously described [6].

### 2.4. Analysis Implementation

Implementation of machine learning algorithms in this study can be broadly divided into two categories based on the way the features are extracted for classification purpose: The rule based group such as decision trees (DT), random forest (RF), support vector machine (SVM), K-Nearest Neighbors (KNN) for which the features were extracted from the EEG and fed into the algorithms manually and the automatic feature selection group such as convolutional neural network (CNN).

#### 2.4.1. Rule-Based Machine Learning Algorithms

Rule-based machine learning (ML) algorithms consist a set of rules and conditions depending on which a decision is made on the category to which the input belongs to. Decision trees (DT) and random forests (RF) are examples in which each node in the tree is a condition whose result enables one to classify as the algorithm progresses along the tree. All machine learning algorithms used are supervised learning algorithms where the target label is already known to the algorithm. DT builds tree-structured models incrementally, as it breaks down the training dataset into smaller subsets [16]. RF takes the majority vote of several decision trees’ prediction which are trained on different parts of the same dataset. SVM creates a hyperplane separating the classes by mapping data to a high dimensional feature space [17]. Based on feature similarity, KNN algorithm classify an object based on majority vote of its neighbors with the object being assigned to the class most common among its K-nearest neighbors [18]. One of the major advantages of rule-based ML algorithms is the use of domain knowledge in terms of selecting meaningful features which can be interpreted with the knowledge specific to the domain in which one is working. Consequently, only the feature vector acts as the input to the machine learning algorithm instead of the entire dataset. Hence, the need for high computational power and storage is eliminated.

For the analysis, 1 min non-overlapping wake intervals were extracted from each 24-h mice EEG files. Each epoch was then filtered into different frequency sub-bands: theta (4–7.5 Hz), alpha (8–12 Hz), sigma (13–16 Hz), beta (16.5–25 Hz) and gamma (30–35 Hz) using a 6th order Butterworth bandpass digital filter. Average power in each frequency sub-band was calculated for each epoch by calculating power using 256 × 60 point, 1-D Discrete Fourier Transform (DFT) and taking its mean. The average power was decibel normalized using the formula

(1) dB=10×log10(activity/baseline).

The need for decibel normalization arises since the comparison is made across frequencies. Decibel normalization is an important step because power amplitude of frequency specific activity generally decreases with increase in frequency, thereby making it difficult to visualize any activity which cause slight changes at higher frequencies [19]. The baseline for decibel normalization was calculated as the average of the first 5 epochs from the mice used in the training dataset. Same baseline values obtained from the training dataset for individual frequency bands were used to normalize the test mice as well. The ratio of alpha:theta power was also calculated as a feature. Feature matrix was normalized to zero mean and unit standard deviation before being fed to the machine learning models. The normalization parameters used, *i.e*. mean and standard deviation for each feature were calculated only for the training dataset and the same was used to normalize the test dataset. These normalization steps were taken to ensure less inter-subject variability and also to account for practical scenarios where the models do not have any prior information regarding the test subjects. Python 3.7 along with Spyder IDE and machine learning tool: scikit-learn was used to implement and test the algorithms. Classification accuracy for ‘K-nearest neighbor’ (KNN) is reported for three different values of ‘K’ 3, 5 and 7. ‘Neural network’ is designed with two hidden layers containing 5 nodes each and ‘Support vector machine’ (SVM) uses Radial basis function (rbf) kernel function. All classification accuracy reported in percentage (%) are given by

(2) Accuracy=(Truepositives+Truenegatives)/Totalobservations.

Data from 9 mice were used in this study with a total of 20 trials, each containing 4 sham mice and 3 mTBI mice in the training set and test set containing 1 sham and 1 mTBI mice. The analysis was also performed with 2 min and 4 min non-overlapping epochs to compare the effect of epoch lengths on the classification accuracy. Though the total duration of EEG recording was 24 h, the number of non-overlapping awake epochs obtained is different among mice as they vary in their sleep cycles. For a mice having frequent transitions between the sleep stages during the period of the recording, the number of awake non-overlapping epochs extracted would be less compared to that of mice having continuous bouts of wakefulness. The number of awake epochs extracted in different mice is tabulated in Table 1.

#### 2.4.2. Convolutional Neural Network (CNN)

While those aforementioned rule-based models give acceptable performance in modeling characteristics of the signal, they still highly depend on handmade features designed to represent data sessions. Moreover, these models tend to skip high-resolution temporal dynamics of data. Convolutional neural networks (CNNs), leveraging shared parameters, have shown to be more effective in capturing these dynamics. These networks are constructed by consecutive neural layers, and typically consist of a number of convolution and pooling layers followed by some dense layers ending with a max pooling layer aimed to produce the final classification label. These structures typically require higher volume of data to achieve better performance in comparison to other machine learning models. Such data-driven property makes CNNs prone to the over-fitting problem where the model tends to perform much better on training, comparing to test data (also known as memorizing data). This lack of generalization problem normally bounds researchers to train networks with a small amount of parameters. Furthermore, different methods have been proposed to train deeper yet more generalized models, namely L1 and L2 regularizations, batch normalization, dropout layers, and more data-side solutions like data augmentation [20,21]. Owing to the ability to efficiently pick up intricate features of data, CNNs have been widely deployed in many complex machine learning tasks from image processing to speech recognition [22,23,24].

**Figure 2:**
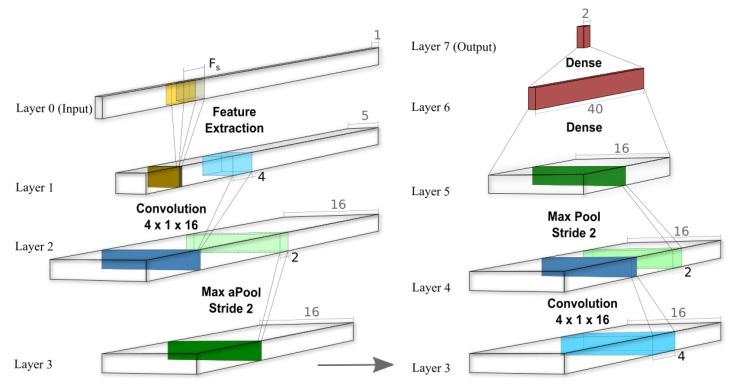
The used convolutional neural network (CNN) architecture. In the feature extraction layer, we are using 1-second windows (containing F_s_ numbers) with overlap of half second extracting mentioned 5 band averages. The extracted features are being fed to 16 1-D convolution filters with kernel of 4, then there is a max pool of stride 2. These Conv-Pool combination repeats one more time, ending with a dense layer of 40 neurons and then the soft-max final layer.

Figure 2 demonstrates our feature extraction and CNN pipeline, with a relatively shallow architecture. As shown (in Layer 1), a one-second feature extraction window slides over the signal with half-second overlapping, extracting those aforementioned 5 average frequency bands. Our network takes these features as input, passing them to 16 filters of one-dimensional convolution layer with kernel size of 4 and Relu activation functions. This convolution layer is followed by a MaxPooling layer with a stride of two, reducing the signal length to half. An identical Conv-Pool combination repeats after that, giving the output to a batch-normalization layer then to 40 neurons of dense layer with L1 regularization equal to 0.001. Here, we use categorical cross-entropy with the Adam optimizer [25] and early stopping of 50 epochs for training. Although other works proposed (for different tasks) deeper networks, avoiding manual feature extraction layers, yet our initial experiments showed leveraging decibel normalization method on the features significantly helps generalize the model [26,27]. A large domain mismatch between train and test sessions makes this normalization method crucial.

## 3. Results and Discussion

**Figure 3:**
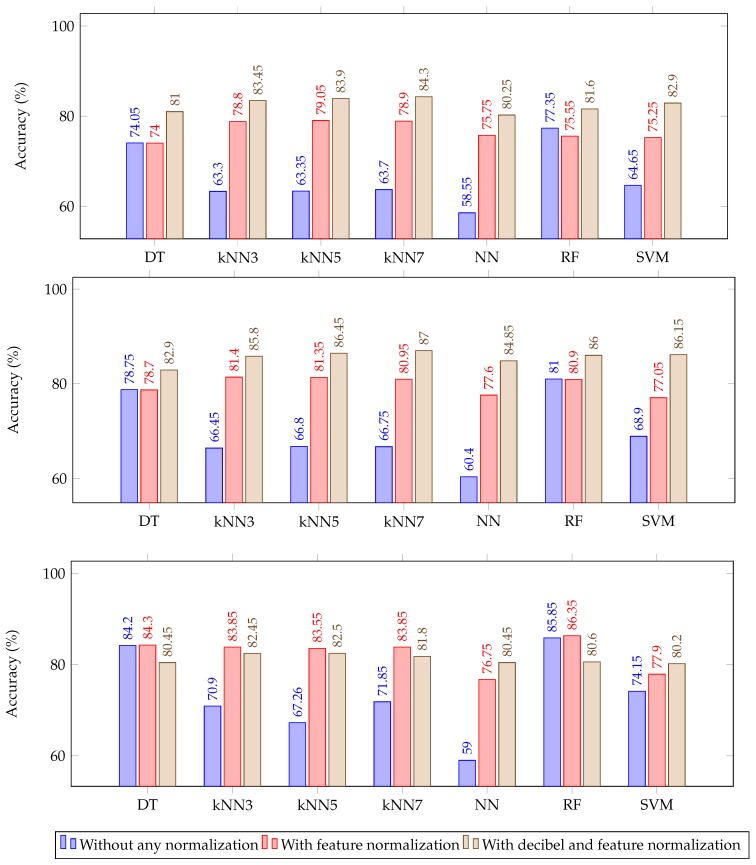
Cross-validation accuracy of various rule based classifiers using different normalization technique for epoch length of 1 min (**top**), 2 min (**middle**) and 4 min (**bottom**).

This section presents a comprehensive analysis of the results obtained for different scenarios considered in previous sections. Each graph in Figure 3 shows classification accuracy obtained in various rule based ML algorithms for three different scenarios of normalization. The blue bars in Figure 3 represent classification accuracy with no normalization techniques. The red bars represent classification accuracy when feature normalization was introduced to normalize feature matrix to zero mean and unit standard deviation before feeding it to the ML algorithms. Whereas the brown bars represent classification accuracy when baseline normalization was carried out using decibel normalization as described in the method section before implementation of feature normalization thereby including both baseline as well as feature normalization. The baseline for decibel normalization considered here is the average of first 5 epochs from the mice used in the training dataset. It is obvious from Figure 3 that normalization plays a significant role in the performance of the deployed ML algorithms as there is considerable increase in the classification accuracy with the introduction of decibel and feature normalization in all the epoch lengths considered.

**Figure 4:**
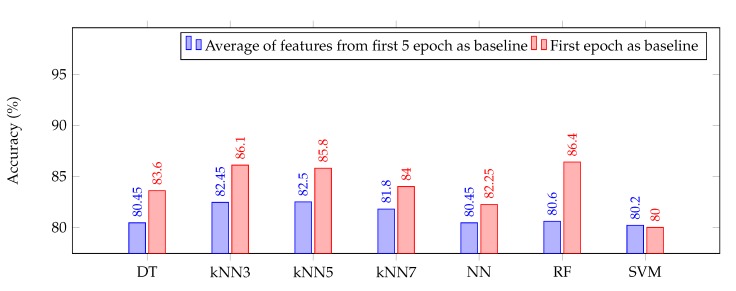
Cross-validation accuracy of various rule based classifiers using different baseline for normalization for epoch length of 4 min.

However, the bottom chart in Figure 3 demonstrating classification accuracy for 4 min epochs, shows a slight decrease in the accuracy in some of the classifiers when decibel normalization was introduced. This dip in accuracy may be due to the sub-optimal choice of the baseline which plays a significant role in the classification accuracy. This was investigated by changing the baseline from an average of features from the first 5 epochs, which effectively is the first 20 min (5 × 4 min) of EEG recording to feature only from first epoch thereby reducing the baseline to first 4 min of recording. This change in baseline yields a clear increase in the performance of all ML algorithms as shown in Figure 4.

**Figure 5:**
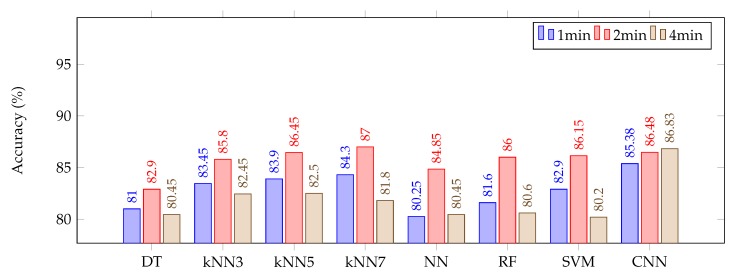
Cross-validation accuracy of various classifiers using different epoch lengths.

There was a similar increase in the classification accuracy when the first 4 min of EEG recording was considered as baseline for 1 min and 2 min epoch classifications as well which is not shown in this paper. Figure 5 demonstrates the results of all models we have evaluated with features from first 5 epochs as baseline in each case. Each bar chart represents the accuracy of one model for three different epoch lengths of 1, 2 and 4 min of wake signals. Results for longer epoch lengths are not presented in this paper since the number of non-overlapping epochs extracted from each mice or the number of data points for our model to train on, will drastically reduce as the length of epoch increases thereby, reducing the accuracy in such cases. We believe the epoch lengths presented in the paper along with number of epochs extracted in each mice, presented in Table 1 is sufficient to show the trend in the accuracy. The accuracies reported are the mean value over all cross-validation experiments leaving two recording sessions aside as test sets. It is clear that for the rule-based models, 2 min is the optimum epoch length. We can observe that for 2 min epoch length, the best result (87%) among rule-based models is achieved by KNN7.

For the CNN model, as mentioned earlier, we trained the network on 20 iterations, putting 10% of data aside for validation. As seen in the chart, CNN generally performs better than rule-based models (unless for 2 min epoch which is still very close to KNN7). We hypothesize that is due to taking high resolution temporal dynamics of the signal into account. The first layer of convolution (with 16 kernels) transforms the input data to a richer representation (in comparison to 5 feature dimension). The max pooling in the following, reduces the resolution by passing only the maximum values. The next convolution-pool pair, enables model to learn more complex behaviours. In the following, dense layer maps all convolved information to a fixed length representation of the data. This layer performs as an embedding for the whole data session that the final classification layer consumes to make the prediction on the class. Another aspect which may be affecting the performance is the amount of epochs with which each model is being trained on. As shown in Table 1, it may be not enough for the rule-based models, while CNN leverages the ability to compare more data. The CNN method is more promising in terms of reliability since there is little difference between different epoch lengths. We also computed the average of variances among all cross-validation experiments for each model (as another measurement of reliability). This value for CNN was 0.92% while those for KNN3, KNN5 and KNN7 were 1.93%, 1.94% and 2.10% respectively. Moreover, we can see that the performance of CNN slightly increases as the epoch length increases, which further confirms its data-driven performance. This is our first attempt of investigating various machine learning approaches to classify TBI and sham using 24-hour recording data from 9 mice. Since CNN is a data-intensive approach, generalization of the model presented in this paper needs to be further investigated and optimized in our future work when additional data are available.

## 4. Conclusions

To the authors’ best knowledge, this is the first study comparing the performance of different ML algorithms to classify mTBI using EEG signals. The accuracy obtained and reported in this paper is higher than those of previous studies [12]. Of note, we report the classification accuracy per epoch unlike other studies which have reported accuracy as the number of total subjects classified correctly to the total number of subjects in the study [12]. Our approach thus gives a better understanding of the performance of ML algorithms in detecting mTBI via EEG. As stated in the results section, CNN outperforms other classical rule-based ML algorithms; however, various parameters such as epoch length of the EEG, pre-processing methods such as independent component analysis (ICA), filtering techniques, sleep stages, and most importantly other normalization methods to reduce inter-subject variability have to be explored. Here, we also propose to use adaptive baseline calculation considering different sections of the EEG recording. Other aspects to enhance the performance of the models could include more domain-specific knowledge that would control for differences in the distribution of sleep vs. wake between individuals. Comparison of ML algorithms based on various computational parameters such as computation time and memory usage have to be investigated to get a much clearer understanding of their performance. For future studies, we intend to explore sleep stages in EEG signals as well and then try to transfer the learned models from mice to human EEG signal domain. 

## Figures and Tables

**Table 1:** Number of 1 min, 2 min and 4 min non overlapping wake epochs in each mouse.

Subjects	1 min	2 min	4 min
Sham102	736	352	168
Sham103	637	275	101
Sham104	922	427	186
Sham107	684	316	146
Sham108	780	364	164
TBI102	901	429	201
TBI103	271	81	16
TBI104	207	61	12
TBI106	458	181	59
